# Berlin Letter

**Published:** 1885-01

**Authors:** 


### Foreign Correspondence.

BERLIN, November 28, 1884.

*Editors Buffalo Medical and Surgical Journal:*

Lisfranc's method of excising the lower end of the rectum in cases of cancerous formations of the anus, seems now to be almost universally employed in the schools of Germany. The idea, when first suggested, was very favorably received, but the large number of fatal results caused it to fall into gradual disuse. Its revival, however, within the last few years, has been regarded with no little interest, from the fact that so much unfavorable criticism had already been passed upon it. Now that Germany has adopted this means of eradicating the cancer, better results might well be looked for; but if the three operations, which I have witnessed, may be taken as an index of its success in this country, the outlook is certainly far from satisfactory.

Of these cases, two resulted in death within forty-eight hours after the operation was performed; the other lingered some two weeks and then died, ulceration having attacked the small part of the deposit which the operator had failed to include in the excision. It the first two cases death was caused by a slight wound of the peritoneum, and, indeed, this has always been regarded as the chief danger attending such an operation. Add to this the great danger of hemorrhage, and we undoubtedly have the formidable obstacles with which the surgeon has to deal. It is usually necessary to remove from two to three inches of the rectum, together with a part of the ischio-rectal fossa, and when working within so limited a space, and at a distance so far from the surface, a wound of the serous membrane is almost inevitable.

The idea, suggested by Denouvilliers, of connecting with the incision drawn around the morbid growth, a deep incision in the middle line of the ischio-rectal fossa extending to the coccyx, enables the operator to work with a somewhat greater degree of certainty; at the same time the necessity of a careful dissection into the ischio-rectal fossa is scarcely benefited by this proceeding, and the danger of hæmorrhage seems to be increased. In order to avoid the risk of bleeding the thermo-cautery is sometimes employed in the deeper dissections, but this can only be used to advantage in the case of fast-growing and vascular tumors. In the ordinary epithelioma, which is by far the most common form of cancer in the region of the anus, the chief objection to excising the lower end of the rectum lies in the fact that the cancer is often found in disconnected parts, and frequently one or more of these deposits is situated above the part excised. A post mortem in one of the above-mentioned cases revealed this fact, and I dare say many of the unsuccessful operations result from the same cause. It is exceedingly dangerous to remove more than about three inches of the rectum, and, therefore, when a cancerous deposit is above this, the operation avails nothing. The fact that the operator is unable to define with any degree of certainty the limits of the cancer, seems to me, in itself, sufficient to condemn the operation.

It would appear that in most instances of cancerous affections of the lower end of the rectum, equal relief might be derived by the operation of colotomy, and with much less risk to the patient. We now know that after successful colotomy, or, indeed, after the formation of an artificial anus in any part of the intestine, the patient's life is not by any means so miserable as might be supposed. The cancer is usually of the less malignant form, and if the irritation, which frequently results from mechanical obstruction, can be avoided, the patient may survive a long time. After excising the lower end of the rectum, if the patient lives, he rarely ever regains control over the fæces, and the danger of a speedy recurrence of the deposit, renders the operation useless except for temporary relief.

Sincerely, G. W. L., Jr.

